# The Hidden Signal: P Wave Morphology and In-Hospital Mortality in Acute Pulmonary Embolism

**DOI:** 10.3390/diagnostics15202636

**Published:** 2025-10-19

**Authors:** Corina Cinezan, Alexandra Manuela Buzle, Maria Luiza Hiceag, Camelia Bianca Rus

**Affiliations:** 1 Department of Medical Disciplines, Faculty of Medicine and Pharmacy, University of Oradea, 410073 Oradea, Romania; buzle.alexandramanuela@student.uoradea.ro; 2 Clinical County Emergency Hospital Bihor, 410169 Oradea, Romania; 3 Doctoral School of Biological and Biomedical Sciences, University of Oradea, 410087 Oradea, Romania; 4 Cardiology Department, Rehabilitation Hospital, 400347 Cluj-Napoca, Romania; maria.luiz.hiceag@elearn.umfcluj.ro; 5 Cardiology Department, Municipal Hospital Aiud, 515200 Aiud, Romania

**Keywords:** pulmonary embolism, P wave morphology, electrocardiography, mortality, risk stratification, prognostic marker

## Abstract

**Background**: Electrocardiographic (ECG) abnormalities are common in acute pulmonary embolism (PE), but the prognostic significance of P wave morphology remains unclear. Early identification of high-risk patients is critical for guiding therapy and monitoring. **Methods**: We retrospectively analyzed 300 patients with confirmed PE. P wave morphology (normal, biphasic, notched, peaked) was evaluated for association with in-hospital mortality using chi-square and logistic regression, adjusted for age, sex, PESI score, and oxygen saturation. **Results**: Mortality differed significantly across P wave groups (χ^2^ = 35.3, df = 3, *p* < 0.001). In univariate analysis, biphasic (OR 15.38, 95% CI 5.02–47.10, *p* < 0.001) and peaked (OR 7.21, 95% CI 2.35–22.10, *p* = 0.001) morphologies were strongly associated with mortality, whereas notched P waves were not (OR 1.44, 95% CI 0.16–12.87, *p* = 0.743). After adjustment, biphasic (OR 14.87, 95% CI 4.77–46.37, *p* < 0.001) and peaked (OR 6.58, 95% CI 2.11–20.53, *p* = 0.001) shapes remained independent predictors. Age, sex, PESI score, and oxygen saturation were not significant in multivariable analysis. **Conclusions**: Biphasic and peaked P wave morphologies on ECG are strong predictors of in-hospital mortality in patients with PE. Routine assessment of P wave shape may provide a simple tool for early risk stratification, warranting validation in prospective cohorts.

## 1. Introduction

Pulmonary embolism (PE) is a potentially life-threatening cardiovascular emergency characterized by acute obstruction of the pulmonary arterial circulation, most commonly due to thromboembolism originating from the deep veins of the lower extremities. PE remains a major cause of morbidity and mortality worldwide, with in-hospital mortality rates ranging from 5% to 15% depending on the severity of presentation and underlying comorbidities [1]. Despite advances in diagnostic imaging and therapeutic strategies, early risk stratification remains a cornerstone of clinical decision-making, as it guides the need for intensive monitoring, advanced therapies, and consideration of thrombolysis or catheter-directed interventions [2].

Several risk stratification tools have been developed, including the Pulmonary Embolism Severity Index (PESI) and its simplified version, which integrate demographic, clinical, and hemodynamic parameters. While these scores are widely validated, they require multiple data points and may not be immediately available at the time of presentation [2]. Consequently, there is continued interest in rapid, inexpensive, and widely accessible markers that could complement established risk models. Among these, the 12-lead electrocardiogram (ECG) represents a ubiquitous, non-invasive, and cost-effective diagnostic tool with the potential to provide prognostic information in patients with acute PE [3,4].

Electrocardiographic abnormalities are frequently observed in the setting of acute PE and are generally attributed to acute right ventricular strain and pressure overload. Well-recognized patterns include sinus tachycardia, right axis deviation, right bundle branch block, T-wave inversion in the right precordial leads, and the so-called S1Q3T3 pattern [5,6]. Although these findings may support the clinical suspicion of PE, they are neither sufficiently sensitive nor specific to establish the diagnosis. However, beyond their diagnostic limitations, ECG changes may hold prognostic value, reflecting the degree of hemodynamic compromise and right ventricular dysfunction—two critical determinants of outcome in PE. Recent studies have demonstrated that combining ECG abnormalities with biomarkers, such as NT-proBNP, enhances prognostication in PE, and that digital ECG-based tools for detecting RV dysfunction are becoming increasingly accurate [5,7,8].

Among the less extensively studied ECG features, P wave morphology has recently attracted considerable attention. The P wave represents atrial depolarization and is influenced by atrial size, conduction pathways, and interatrial pressure changes. Alterations in P wave configuration may reflect elevated right atrial pressures, atrial conduction delays, or electrical remodeling in response to acute hemodynamic stress. In particular, morphologies such as biphasic, notched, or peaked P waves may indicate significant right atrial overload and mechanical stress secondary to acute PE. While abnormalities in P wave duration and dispersion have been linked to atrial arrhythmias and adverse cardiovascular outcomes in other settings, the potential prognostic role of P wave morphology in acute PE has not been systematically examined [9,10].

Existing studies on ECG prognostic markers in PE have largely focused on repolarization changes, QRS axis shifts, and arrhythmic complications. Only limited data are available on the contribution of atrial depolarization abnormalities to clinical outcomes in this population. Given the simplicity of P wave assessment and its potential as a bedside marker of right atrial strain, elucidating its prognostic significance could provide clinicians with a rapid and widely accessible tool for risk stratification [11].

In this context, we hypothesized that specific P wave morphologies are associated with increased in-hospital mortality in patients with acute PE. To test this hypothesis, we conducted a retrospective study of 300 patients with confirmed PE, systematically evaluating P wave configurations on admission ECG and their association with clinical outcomes. Both univariate and multivariable logistic regression analyses were performed to determine whether P wave morphology independently predicts mortality, after adjusting for established risk factors, including age, sex, PESI score, and oxygen saturation.

It is important to acknowledge that the diagnostic landscape of acute PE has shifted in recent years. While traditionally considered an emergency-room diagnosis [12]. PE is now increasingly identified in internal medicine and geriatric wards, often in patients with multiple comorbidities and atypical presentations. Consequently, both diagnostic and prognostic tools initially developed for emergency settings must now be validated in this broader, real-world clinical context [13].

## 2. Methods

### 2.1. Study Design and Population

We performed a retrospective observational study including 300 consecutive patients admitted with a confirmed diagnosis of acute pulmonary embolism (PE) between January 2022 and August 2025 at the Cardiology Department, Clinical County Emergency Hospital, Bihor. The diagnosis of PE was confirmed by computed tomography pulmonary angiography (CTPA) in accordance with current European Society of Cardiology (ESC) guidelines.

Inclusion criteria were: adults (≥18 years) admitted with confirmed diagnosis of acute PE, availability of a standard 12-lead ECG at admission with analyzable P wave morphology. ECG interpretation was independently performed by two cardiologists blinded to patient outcomes. In case of disagreement, a third senior cardiologist adjudicated the final morphology classification.

Exclusion criteria were: missing or poor-quality ECG that prevents reliable P wave morphology analysis or other ECG changes: atrial enlargement that may confound P wave morphology interpretation, permanent pacemaker, bundle branch blocks, atrial fibrillation/flutter at admission ECG (since these alter P wave morphology), junctional rhythm, atrioventricular and sinoatrial block. Other exclusion criteria included prior chronic pulmonary hypertension, congenital heart disease, heart failure, advanced cardiovascular, hepatic, or chronic kidney disease requiring hemodialysis, dementia, and terminal illnesses. Patients with submassive or massive PE who received immediate thrombolysis or surgical embolectomy before ECG could be obtained, those with recurrent PE during admission (difficult to assign ECG changes to index event), lack of complete clinical data (PESI score, oxygen saturation, demographics) were excluded, too.

Upon admission, patients signed an informed consent stating that they agreed to their medical data being used for scientific or educational purposes in an anonymous format.

### 2.2. Clinical Data Collection

Baseline demographic and clinical data, including age, sex, and vital signs at presentation, were extracted from medical records. The Pulmonary Embolism Severity Index (PESI) score was calculated for each patient based on established criteria. Arterial oxygen saturation on room air was recorded at admission. The primary outcome was in-hospital mortality, defined as death from any cause during the index hospitalization.

### 2.3. Electrocardiographic Assessment

Standard 12-lead ECGs were obtained at admission and independently analyzed by two experienced cardiologists blinded to patient outcomes. P wave morphology was classified in lead II as follows:

- Normal: Smooth, monophasic deflection of typical duration and amplitude.
- Biphasic: Initial positive deflection followed by a terminal negative component.
- Notched: Double-peaked P wave with two positive deflections.
- Peaked: Sharply pointed P wave exceeding normal amplitude thresholds.

**Figure 1:**
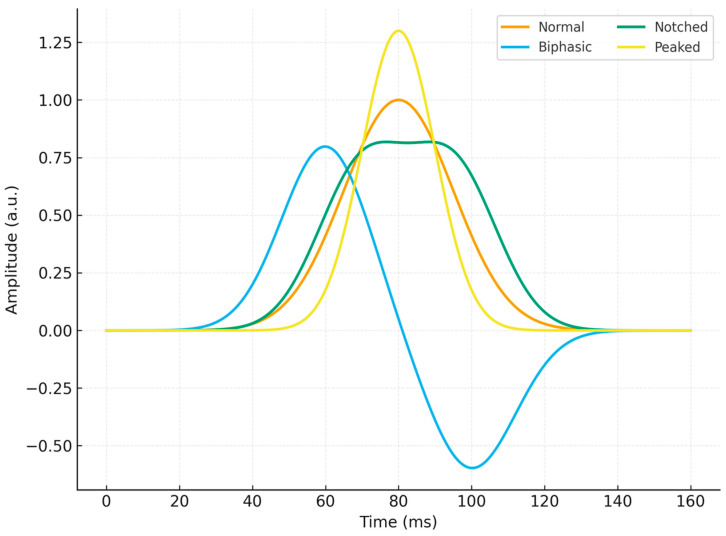
Representative P wave morphologies (normal, biphasic, notched, peaked) as observed in lead II.

Representative examples of each P wave morphology are illustrated in Figure 1.

### 2.4. Statistical Analysis

Continuous variables were expressed as mean ± standard deviation (SD) or median with interquartile range (IQR), depending on distribution. Categorical variables were presented as frequencies and percentages. Group differences in categorical outcomes were compared using the chi-square test or Fisher’s exact test, as appropriate.

Univariate logistic regression was used to assess the association between P wave morphology and in-hospital mortality, with normal morphology serving as the reference category. Multivariable logistic regression was subsequently performed, adjusting for clinically relevant covariates: age, sex, PESI score, and oxygen saturation. Results were expressed as odds ratios (OR) with corresponding 95% confidence intervals (CI).

Model fit was assessed using the likelihood ratio test, and multicollinearity was excluded by variance inflation factor analysis. Statistical significance was set at a two-sided *p*-value < 0.05. All analyses were performed using Python (pandas, statsmodels, scipy, matplotlib).

## 3. Results

### 3.1. Baseline Characteristics

A total of 300 patients with acute pulmonary embolism were included. The mean age was 64 years, and 52% were male. The distribution of P wave morphology at admission was as follows: normal in 59.3%, biphasic in 13.0%, notched in 8.3%, and peaked in 19.3%. The median PESI score was 86, and the mean oxygen saturation on admission was 92%. Overall, in-hospital mortality occurred in 28 patients (9.30%).

Table 1 summarizes baseline patient characteristics by P wave morphology.

**Figure 2:**
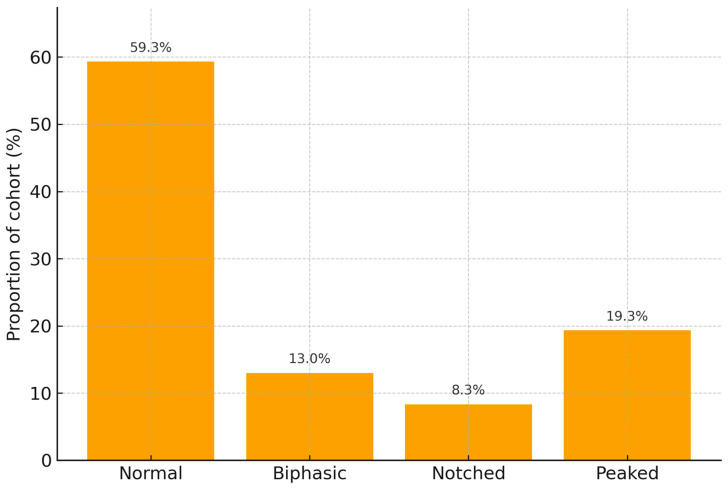
Distribution of P-wave morphologies among the 300 patients.

Figure 2 represents the distribution of P-wave morphologies among the 300 patients.

### 3.2. Association Between P Wave Morphology and Mortality

**Figure 3:**
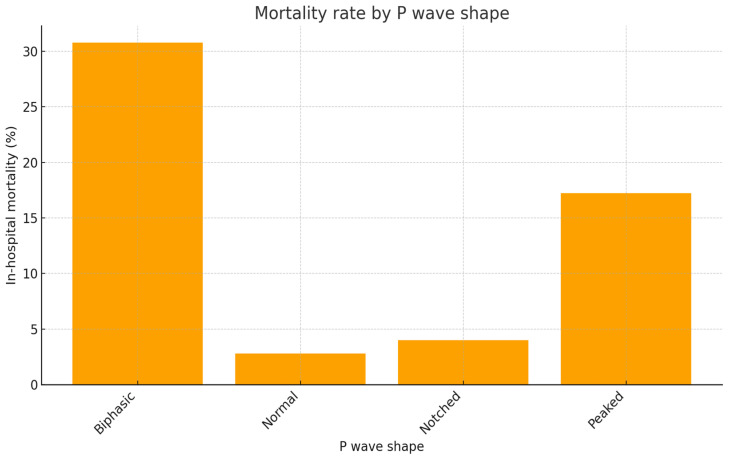
In-hospital mortality rates across P wave categories.

Mortality rates varied significantly across P wave categories (χ^2^ = 35.3, df = 3, *p* < 0.001). Patients with biphasic and peaked P waves had the highest in-hospital mortality, whereas those with notched P waves did not differ substantially from the normal group. Figure 3 illustrates in-hospital mortality rates across P wave categories.

### 3.3. Univariate Logistic Regression

Compared with patients with normal P waves, biphasic morphology was associated with a 15-fold higher risk of mortality (OR 15.38, 95% CI 5.02–47.10, *p* < 0.001). Peaked morphology also showed a strong association (OR 7.21, 95% CI 2.35–22.10, *p* = 0.001). Notched morphology was not significantly associated with in-hospital mortality (OR 1.44, 95% CI 0.16–12.87, *p* = 0.743).

Table 2 presents univariate logistic regression results.

### 3.4. Multivariable Logistic Regression

After adjusting for age, sex, PESI score, and oxygen saturation, biphasic (OR 14.87, 95% CI 4.77–46.37, *p* < 0.001) and peaked (OR 6.58, 95% CI 2.11–20.53, *p* = 0.001) morphologies remained independent predictors of in-hospital mortality. Notched morphology was not associated with mortality (OR 1.34, 95% CI 0.15–12.23, *p* = 0.793). None of the covariates (age, sex, PESI score, oxygen saturation) was independently significant in the multivariable model.

Table 3 details multivariable logistic regression outcomes.

**Figure 4:**
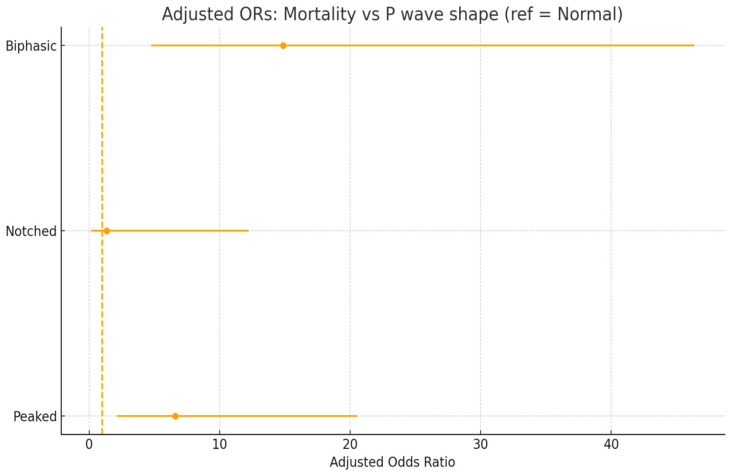
The figure displays adjusted odds ratios for mortality associated with abnormal P wave morphologies.

Figure 4 shows adjusted odds ratio for mortality with abnormal P wave morphologies.

### 3.5. Effect Sizes and Clinical Interpretation

In univariate logistic regression, biphasic P waves were associated with a markedly increased risk of in-hospital mortality (OR 15.38, 95% CI 5.02–47.10), corresponding to a mortality rate of 30.8% compared with only 2.8% in patients with normal P waves. Peaked P waves were also strongly associated (OR 7.21, 95% CI 2.35–22.10), with a mortality rate of 17.2%. The absolute risk difference between biphasic and normal morphology was 28.0%, while that between peaked and normal was 14.4%. Notched P waves, although associated with a numerically higher mortality rate (4.0%), did not differ significantly from normal P waves. (OR 1.44, 95% CI 0.16–12.87).

In the adjusted model, biphasic morphology remained associated with nearly a 15-fold increase in odds of death, and peaked morphology with a 6.5-fold increase, independent of age, sex, PESI score, and oxygen saturation. Importantly, the wide confidence intervals reflect the relatively small subgroup sample sizes, particularly for the notched group, and should be interpreted with caution.

The effect sites of P wave morphology on in-hospital mortality is shown in Table 4.

### 3.6. Subgroup Analysis

When stratified by PESI class (I–III vs. IV–V), biphasic and peaked P-wave morphologies remained significantly associated with in-hospital mortality in both strata (*p* < 0.01). The effect was more pronounced in high-risk patients (PESI IV–V), where biphasic morphology predicted a 38.5% mortality rate compared with 3.6% among normal P waves (*p* < 0.001).

Table 5 presents the subgroup analysis of in-hospital mortality stratified by Pulmonary Embolism Severity Index (PESI) risk class. Patients were divided into low/moderate (Classes I–III) and high-risk (Classes IV–V) categories. Mortality rates were compared using the chi-square test. Both biphasic and peaked P-wave morphologies remained significantly associated with mortality across risk strata, supporting the robustness of the findings.

## 4. Discussion

### 4.1. Main Findings

In this retrospective study of 300 patients with acute pulmonary embolism (PE), we found that P wave morphology on admission ECG carried important prognostic information. Specifically, biphasic and peaked P wave morphologies were strongly and independently associated with increased in-hospital mortality, even after adjustment for established clinical predictors such as age, sex, Pulmonary Embolism Severity Index (PESI) score, and oxygen saturation. In contrast, notched P waves did not demonstrate a significant association with outcomes. These findings highlight the potential utility of simple ECG assessment as a rapid, accessible adjunct to established risk stratification tools in the management of acute PE.

### 4.2. Comparison with Existing Literature

The prognostic role of electrocardiographic abnormalities in acute PE has been investigated primarily in relation to right ventricular strain markers. Patterns such as the S1Q3T3 sign, T-wave inversion in precordial leads, right bundle branch block, and ST-segment changes have been repeatedly reported as indicators of adverse prognosis. However, the clinical utility of these markers has been limited by inconsistent reproducibility and low sensitivity [14,15].

Much less attention has been directed toward P wave morphology. Prior studies have described abnormal P wave configurations in the context of atrial enlargement, conduction disturbances, and pulmonary hypertension. In particular, peaked P waves (“P pulmonale”) have long been associated with right atrial overload, whereas notched P waves have been linked to intra-atrial conduction delay. Biphasic P waves, especially in lead II or V1, typically reflect interatrial conduction block or marked atrial pressure changes [16].

Our findings are consistent with earlier observations that acute increases in right atrial pressure due to PE can alter atrial depolarization, producing peaked or biphasic P waves. Geibel et al. [17] observed that specific ECG abnormalities, including those related to atrial depolarization, were significant predictors of in-hospital mortality, supporting the relevance of P wave morphology in PE prognosis.

Kukla et al. [4] found that specific ECG abnormalities, including P wave alterations, predict adverse outcomes, underscoring the prognostic value of P wave morphology and its association with a higher mortality risk in PE.

Chen et al. [18] highlighted that P-wave parameters reflect atrial conduction and morphology, which are critical for arrhythmia risk and prognosis.

Furthermore, our findings complement those of Choi et al., who validated ECG-derived digital markers of right ventricular dysfunction with high discriminative performance [7]. Raghubeer et al. [19] demonstrated that certain ECG abnormalities are valuable predictors of in-hospital mortality in PE, supporting the rationale for exploring P-wave features as prognostic markers.

Nillson et al. [5] observed that ECG abnormalities are correlated with elevated NT-proBNP, emphasizing the link between ECG features and myocardial strain in patients with PE. Additionally, Jiao et al. demonstrated that ECG abnormalities together with biomarkers could predict in-hospital adverse events in PE [20].

While these studies focus more on repolarization indices, right axis deviations, or composite digital markers, our study adds by specifically evaluating P-wave morphology as a potentially simple and specific predictor.

However, to our knowledge, no prior large-scale study has systematically assessed whether these specific morphologies predict mortality in acute PE. The strong associations observed in our cohort suggest that P wave analysis—often overlooked in clinical practice—may yield valuable prognostic information.

### 4.3. Pathophysiological Considerations

The mechanisms underlying the prognostic impact of abnormal P wave morphologies in acute PE are likely multifactorial:

1. Right atrial overload and stretch: sudden increases in pulmonary arterial pressure due to thromboembolic obstruction elevate right ventricular afterload. This leads to right atrial pressure elevation, which is reflected on the surface ECG as peaked or biphasic P waves. The severity of such atrial strain may directly correlate with hemodynamic compromise and, consequently, increased risk of death [21].
2. Electrical conduction abnormalities: abnormal P waves may also reflect delayed interatrial conduction or anisotropy of atrial depolarization. This electrical instability could predispose patients to atrial arrhythmias, which are known to complicate the course of PE and worsen prognosis [22].
3. Indirect marker of right ventricular dysfunction: since right atrial pressure is closely tied to right ventricular function, abnormal P wave morphologies may act as a surrogate for right ventricular strain, a well-established determinant of PE-related mortality [21].

The distinction between biphasic and peaked P waves is of particular interest. Biphasic morphologies may indicate more profound disruption of interatrial conduction, while peaked P waves primarily reflect pressure overload [23]. The independent association of both morphologies with mortality in our study reinforces the notion that they reflect distinct yet complementary aspects of acute right atrial stress.

Effect size commentary

The magnitude of the effect observed for biphasic and peaked P waves is clinically striking. An almost 15-fold increase in mortality risk for biphasic morphology highlights that this is not merely a statistical association but a potentially powerful bedside prognostic marker. The absolute risk increase of nearly 30% underscores its clinical relevance. Similarly, the 6.5-fold increased odds of death in patients with peaked morphology indicate that this finding should not be overlooked during routine ECG interpretation.

It is noteworthy that these associations persisted even after adjustment for PESI score and oxygen saturation, suggesting that P wave morphology may capture aspects of acute right atrial and ventricular strain not fully reflected by traditional risk scores. This aspect strengthens its potential role as a complementary marker for rapid risk stratification.

Nevertheless, the wide confidence intervals, particularly in the biphasic group, suggest that caution is required in extrapolating these results to all patient populations. Larger prospective cohorts are needed to refine the precision of effect size estimates and confirm reproducibility.

### 4.4. Clinical Implications

Our findings have several potential clinical applications:

1. Rapid bedside risk stratification: unlike imaging modalities or biomarker assays, ECG is universally available, non-invasive, and inexpensive. Recognition of biphasic or peaked P waves at presentation may provide clinicians with an immediate signal of elevated risk.
2. Adjunct to established scores: while the PESI score and its simplified version are validated tools, they require integration of multiple variables and may not be readily available at the bedside. Incorporating P wave morphology could refine these scores and improve their predictive accuracy.
3. Guidance for monitoring and therapy: patients identified as high-risk based on P wave abnormalities may warrant closer hemodynamic monitoring, earlier echocardiographic assessment of right ventricular function, and consideration of advanced therapies such as systemic thrombolysis or catheter-based interventions.
4. Educational reinforcement: given that P wave morphology is often underemphasized in routine ECG interpretation, our findings highlight the need for clinicians to re-engage with basic ECG principles when evaluating patients with acute PE.

Compared with existing prognostic tools such as the simplified PESI (sPESI), the P wave morphology approach offers a rapid and low-cost alternative. While our data did not include the full set of variables required for PATHOS or biomarker-based models, P wave morphology may complement sPESI by capturing acute right atrial strain, which is not represented in standard clinical parameters [24].

### 4.5. Strengths of the Study

Several aspects strengthen the validity of our results. First, the study analyzed a relatively large cohort of 300 patients with systematically confirmed PE, providing adequate power to detect significant associations. Second, ECG interpretation was performed independently by two cardiologists blinded to outcomes, reducing the risk of observer bias. Third, we used multivariable logistic regression to adjust for established clinical risk factors, ensuring that observed associations were independent of confounding variables. Finally, the outcome of in-hospital mortality is robust, clinically relevant, and unlikely to be subject to misclassification.

### 4.6. Limitations

Our study also has important limitations. First, its retrospective design introduces the possibility of selection bias and unmeasured confounding. For example, we did not systematically include echocardiographic parameters of right ventricular function, which are important prognostic markers. Second, while ECG analysis was standardized, intra- and inter-observer variability may still influence classification of P wave morphology. Although ECG interpretation was performed by trained cardiologists, some inter-observer variability, particularly in differentiating between biphasic and notched P waves, may have influenced classification accuracy. This variability could modestly reduce reproducibility compared with composite clinical scores based on objective anamnestic and bedside measured parameters, like cancer history, heart rate, systolic blood pressure, arterial O_2_ saturation. Third, the number of patients with notched P waves was relatively small, which may have limited our ability to detect significant associations in this subgroup. Fourth, our analysis was restricted to in-hospital mortality, and long-term outcomes were not assessed. Fifth, we studied patients from a single center, which may limit generalizability. Prospective multicenter validation studies are warranted to confirm these findings.

Unlike some recent works [5,7], our study did not incorporate echocardiographic and laboratory parameters (such as troponin, NT-proBNP, and D-dimer) were not included in this analysis due to incomplete data availability in a subset of patients. Incorporating these markers in future studies may provide a more integrated prognostic model combining electrical, biochemical, and structural information. Furthermore, the absence of laboratory biomarkers such as troponin prevented a direct comparison with the PATHOS score, which incorporates cardiac injury markers into risk stratification.

The observed in-hospital mortality in our PESI Class III subgroup (9.3%) slightly exceeded the expected 3.2–7.1% range reported in the 2019 ESC/ERS guidelines. This discrepancy may relate to unmeasured confounders (delayed presentation, unrecorded right-ventricular dysfunction, or comorbidities) and reflects the real-world heterogeneity of our retrospective cohort.

## 5. Conclusions

In this retrospective study of 300 patients with acute pulmonary embolism, we found that specific P wave morphologies on admission ECG carry strong prognostic significance. Biphasic and peaked P waves were independently associated with markedly increased in-hospital mortality, even after adjustment for established clinical predictors such as age, sex, PESI score, and oxygen saturation. In contrast, notched P waves did not predict adverse outcomes in our cohort. These findings suggest that simple evaluation of P wave morphology, an often-overlooked aspect of the ECG, may serve as a rapid, inexpensive, and universally available adjunct for early risk stratification in acute PE. Incorporating P wave assessment into clinical practice could enhance the identification of high-risk patients who may benefit from intensified monitoring or advanced therapeutic strategies. Prospective, multicenter studies are warranted to validate these results and explore the integration of P wave morphology into existing risk prediction models.

Considering the promising results of AI-driven ECG analysis (Gokhale et al. [8]) and smartphone-based digital biomarker detection (Choi et al. [7]), there is scope to develop automated ECG-based algorithms that include P-wave morphology, potentially enhancing sensitivity and enabling real-time risk stratification even in resource-limited settings.

### Future Directions

Future research should aim to validate these findings in larger, prospective cohorts, ideally with standardized integration of ECG, echocardiographic, and biomarker data. Exploring the interaction between P wave morphology and right ventricular dysfunction on imaging could clarify mechanistic pathways. In addition, evaluating whether incorporation of P wave morphology improves the discriminatory performance of existing prognostic scores would be clinically valuable. Finally, studies addressing the long-term prognostic significance of P wave abnormalities in PE survivors may further expand the relevance of this marker [17,25].

## Figures and Tables

**Table 1:** Baseline characteristics of patients with pulmonary embolism according to P wave morphology.

P Wave Shape	*n*	Age (Years, Mean ± SD)	Male Sex *n* (%)	PESI (Median, IQR)	Oxygen Saturation (%)	Mortality *n* (%)
Normal	178	65.2 ± 11.0	98 (55.1%)	84 (72–98)	92.3 ± 4.0	5 (2.8%)
Biphasic	39	65.1 ± 13.3	24 (61.5%)	85 (74–96)	93.1 ± 3.9	12 (30.8%)
Notched	25	63.2 ± 14.1	13 (52.0%)	89 (79–97)	91.2 ± 4.1	1 (4.0%)
Peaked	58	62.3 ± 11.9	32 (55.2%)	88 (72–100)	92.8 ± 4.3	10 (17.2%)
*p*-value	-	0.324	0.870	0.691	0.246	<0.001

Continuous variables were compared using one-way ANOVA (F-test). No significant differences were found among groups (*p* > 0.05 for all).

**Table 2:** Univariate logistic regression of P wave morphology and in-hospital mortality. Odds ratios (OR) and 95% confidence intervals (CI) are shown. Reference category: Normal P wave morphology. Statistical significance defined as *p* < 0.05.

P Wave Shape	Odds Ratio (OR)	95% CI	*p*-Value
Biphasic	15.38	5.02–47.10	<0.001
Notched	1.44	0.16–12.87	0.743
Peaked	7.21	2.35–22.10	0.001

**Table 3:** Multivariable logistic regression for predictors of in-hospital mortality.

Predictor	Adjusted OR	95% CI	*p*-Value
Biphasic P wave	14.87	4.77–46.37	<0.001
Notched P wave	1.34	0.15–12.23	0.793
Peaked P wave	6.58	2.11–20.53	0.001
Age	0.97	0.94–1.01	0.154
Male sex	1.94	0.78–4.82	0.155
PESI score	1.02	0.99–1.04	0.197
Oxygen saturation	1.03	0.93–1.15	0.573

**Table 4:** Effect sizes of P wave morphology on in-hospital mortality.

P Wave Shape	Mortality n/N (%)	Absolute Risk Difference vs. Normal	Univariate OR (95% CI)	Adjusted OR (95% CI)
Normal	5/178 (2.8%)	Reference	Reference	Reference
Biphasic	12/39 (30.8%)	+28.0%	15.38 (5.02–47.10)	14.87 (4.77–46.37)
Peaked	10/58 (17.2%)	+14.4%	7.21 (2.35–22.10)	6.58 (2.11–20.53)
Notched	1/25 (4.0%)	+1.2%	1.44 (0.16–12.87)	1.34 (0.15–12.23)

**Table 5:** Subgroup Analysis of In-Hospital Mortality by P-Wave Morphology.

P Wave Morphology	Mortality % (PESI I–III)	*p* Value	Mortality % (PESI IV–V)	*p* Value
Normal	3.6%	—	9.8%	—
Biphasic	18.2%	0.014	38.5%	<0.001
Peaked	9.1%	0.037	26.9%	0.002
Notched	4.5%	0.712	8.0%	0.655

## Data Availability

The raw data supporting the conclusions of this article will be made available by the authors on request. The data are not publicity available due to privacy reasons.
